# Emotional Experiences Predict the Conversion of Individuals with Attenuated Psychosis Syndrome to Psychosis: A 6-Month Follow up Study

**DOI:** 10.3389/fpsyg.2016.00818

**Published:** 2016-06-01

**Authors:** Fa Zhan Chen, Yi Wang, Xi Rong Sun, Yu Hong Yao, Ning Zhang, Hui Fen Qiao, Lan Zhang, Zhan Jiang Li, Hong Lin, Zheng Lu, Jing Li, Raymond C. K. Chan, Xu Dong Zhao

**Affiliations:** ^1^Pudong New Area Mental Health Center, Tongji University School of MedicineShanghai, China; ^2^Department of Psychosomatic Medicine, East Hospital, Tongji University School of MedicineShanghai, China; ^3^Neuropsychology and Applied Cognitive Neuroscience Laboratory, Key Laboratory of Mental Health, Institute of Psychology, Chinese Academy of SciencesBeijing, China; ^4^Psychological Health Education and Counseling Center, Tongji UniversityShanghai, China; ^5^Department of Clinical Psychology, Nanjing Brain Hospital, Nanjing Medical UniversityNanjing, China; ^6^Mental Health Center, West China Hospital, Sichuan UniversityChengdu, China; ^7^Department of Clinical Psychology, Beijing Anding Hospital, Capital Medical UniversityBeijing, China; ^8^Department of Clinical Psychology, Peking University Sixth HospitalBeijing, China; ^9^Department of Psychiatry, Tongji Hospital, Tongji University School of MedicineShanghai, China; ^10^Department of Psychiatry, First Affiliated Hospital of Chongqing Medical UniversityChongqing, China

**Keywords:** emotional experiences, Attenuated Psychosis Syndrome, prediction, conversion, psychosis

## Abstract

The present study explored the conversion rate in individuals with Attenuated Psychosis Syndrome (APS) and potential predictor for transition in mainland China. Sixty-three participants identified as APS were followed up 6 months later. The results showed that 17% of individuals with APS converted to full-blown psychosis. The converters exhibited significantly poorer emotional experience and expression than the non-converters at baseline. A further binary logistic regression analysis showed that emotional experience could predict the transition (Wald = 4.18, *p* = 0.041, 95% CI = 1.04~6.82). The present study suggests an important role of emotional processing in the prediction of the development of full-blown psychosis.

## Introduction

The Attenuated Psychosis Syndrome (APS) is a clinical condition associated with a significantly higher risk of psychotic disorders. It has been listed as a new category in the appendix (Section 3) as a condition for further study in the *Diagnostic and Statistical Manual of Mental Disorders, Fifth Edition* (DSM-5; American Psychiatric Association, 2013). According to DSM-5, APS was diagnosed when the attenuated positive risk symptom(s) (1) were present at least once a week in the past month, (2) had begun or worsened in the past year, (3) was sufficiently distressing and disabling to the individual to warrant clinical attention, (4) could not be better explained by another mental disorder and was not attributable to the physiological effects of a substance or another medical condition, and (5) did not match with any psychotic disorder. However, the majority of individuals with APS would not convert to frank psychosis. In a meta-analysis based on 2500 individuals with clinical high risk (Fusar-Poli et al., 2012a), researchers found the mean conversion rates to full-blown psychosis was 22, 29, and 36% after 1, 2, and 3 years, respectively. However, the overall conversion rate of APS to full-blown psychosis was just around 34.9% over a 10-year period (Nelson et al., 2013). Such a high proportion of “false positives” suggests a need for improved prediction indicators in addition to the presence of positive symptoms reported by the APS. Recent empirical findings have shown that symptoms severity, negative symptoms in particular (Demjaha et al., 2012; Piskulic et al., 2012; Nelson et al., 2013; Valmaggia et al., 2013), subthreshold disorders of thought content (Nelson et al., 2013; DeVylder et al., 2014; Kantrowitz et al., 2014), and emotional problems in prodromes (Corcoran et al., 2015) could increase the risk for the transition of psychosis. Valmaggia et al. (2013) suggested that symptomatic clustering predicts prognosis better than individual symptoms in the at-risk mental state for psychosis. Several clinical models also have been proposed to further increase the validity of prediction of transition to psychosis in the APS group (Corcoran et al., 2015; Morrison et al., 2015; van Donkersgoed et al., 2015). For example, a study demonstrated that profound deficits in emotion recognition exist in at-risk patients prior to schizophrenia onset (Corcoran et al., 2015). Therefore, an optimal model combining both emotion recognition values and suprathreshold negative symptoms was strongly encouraged to predict the transition in individuals at clinical high risk for schizophrenia. In a summary, there is a clear need for better prediction models that can be used to help clinicians identify a subgroup of subjects that will benefit most from preventive interventions (Fusar-Poli et al., 2012b).

Moreover, the Structured Interview for Psychosis-Risk Syndromes (SIPS) is believed to be the most popular tool for diagnosing individuals at high risk of psychosis (Miller et al., 2003; McGlashan et al., 2010). However, only attenuated positive symptoms were adopted for diagnostic criteria for APS in DSM-5. Some researches have found that prediction algorithms combining 2 or 3 of these variables in SIPS resulted in dramatic increases in positive predictive power compared with the attenuated positive symptoms criteria alone (Cannon et al., 2008; Chuma and Mahadun, 2011). Unfortunately, the predictive value of the deep attenuated symptom items based on the SIPS was rarely explored.

In the present study, we explored the transition rate of individuals with APS in a Chinese sample and a *Post Hoc* method was used to examine possible prediction effects of emotional experience items on psychosis at a 6 month interval.

## Material and methods

### Participants

This study was conducted from November 1^st^, 2012 to June 30^th^, 2013 in seven research sites distributed in mainland China, including six psychiatric hospitals/units (Nanjing Brain Hospital, Nanjing Medical University; West China Hospital, Sichuan University; Beijing Anding Hospital, Capital Medical University; Peking University Sixth Hospital; Shanghai Pudong New Area Mental Health Center; First Affiliated Hospital of Chongqing Medical University) and one mental health & counseling center of Tongji University, Shanghai. At baseline, we recruited 63 individuals [30 females, mean age (s.d.) = 21.9(4.5)] with APS, diagnosed by the clinical psychiatrists or psychologists. All subjects were help-seekers who were referred to the researchers after being diagnosed with a potential risk for psychosis by the doctors or the counselors/psychologists. The inclusion criteria were: (1) meeting the criteria for APS in DSM-5, as assessed with *the Structured Interview for Psychosis-Risk Syndromes (SIPS;* (McGlashan et al., 2010); (2) age between 14 to 30 years old; (3) individuals below 18 years of age had to be accompanied by either a parent or legal guardian; and (4) understanding the aims of the study and signing the consent form. The exclusion criteria included: (1) a history of psychotic disorders; (2) serious risk of harm to self or others; (3) significant chronic medical or neurological conditions; and (4) mental retardation. The study protocol was approved by the Research Ethics Committees of these centers.

### Measures and procedure

#### Clinical assessment

The Structured Interview for Psychosis-Risk Syndromes (SIPS) and the Scale of Psychosis-Risk Symptoms (SOPS, thus SIPS/SOPS; Miller et al., 2003; McGlashan et al., 2010; Chinese version Zheng et al., 2012) were used to determine whether the subjects met the criteria for a putatively psychosis risk status or the Presence of Psychotic Syndrome (POPS). There are 19 attenuated symptoms items in total and consisting of four major symptom dimensions on the SOPS: positive symptoms (5 items), negative symptoms (6 items), disorganized symptoms (4 items), and general symptoms (4 items). Individual items are rated from 0 (absent) to 6 (severe and psychotic), with 3–5 indicating a psychosis risk symptoms. All interviewers received professional training for SIPS prior to the study, and assessed a standard patient with APS through a video system. The intra-class coefficient (ICC) was found to be 0.97 for the SIPS.

Social function was examined using the Global Assessment of Functioning (GAF; Jones et al., 1995), which is a single rating scale for evaluating an individual's psychological, social, and occupational functioning.

The Montgomery-Åsberg Depression Rating Scale (MADRS; Montgomery and Asberg, 1979) was used to assess the depressive symptoms. The questionnaire includes 10 items. Each item is evaluated on a 7-point scale through a face-to-face interview. High scores on the questionnaire indicate high-level depression. All interviewers received professional training for MADRS prior to the study, and assessed a standard patient through a video system. The ICC was found to be 0.95 showing excellent Inter-rater reliability for the MADRS.

#### Follow-up at 6 months

Of the 63 participants, 47 of them were followed up and finished the re-assessment 6 months later. Participants who met the criteria of a psychotic disorder based on the SIPS/SOPS interview were considered as converters.

### Data analysis

We calculated the transition rate at 6-month follow-up. We also compared the SIPS/SOPS scores at baseline between the converters and non-converters using *Post Hoc* Multiple comparisons (LSD method) in the SPSS v20.0, *p* < 0.05 was set as the threshold for significance. Finally, we adopted the binary logistic regression analysis to examine the potential prediction effect of the SIPS/SOPS scores at baseline for the conversion.

## Results

Among the 47 individuals we followed-up, eight of them converted (including 3 females and 5 males), 18 of them maintained the APS state and 21 of them recovered showing scores on any items less than 3. The transition rate was 17% (8/47). Then, we described the scores on the attenuated symptoms on SOPS, as well as the GAF and depressive symptoms at both the baseline and follow-up (Table 1). The comparisons between converters and non-converters showed significant differences at baseline on the item 3 and item 4 of the negative symptoms assessed by SOPS, which are emotional expression and emotional experience (*p'*s < 0.01). They also showed higher scores on item D1 (odd behavior of appearance) and D3 (trouble with focus and attention; *p'*s < 0.05). In order to examine the prediction effects of these two negative symptoms items of the SOPS, a further binary logistic regression analysis was conducted. The results showed that emotional experience (item N4) could predict the transition (Wald = 4.18, *p* = 0.041, 95%CI = 1.04~6.82).

**Table 1:** **Description of attenuated symptoms and related clinical manifestations at baseline and 6-month follow-up interval (Mean ± SD)**.

**SOPS**	**Baseline**			***F***	***p***	**Follow-up**		***F***	***p***
	**APS (*n* = 63)**	**APS-nc (*n* = 39)**	**APS-c (*n* = 8)**			**APS-nc (*n* = 39)**	**APS-c (*n* = 8)**		
P1(unusual thought content/delusional ideas)	2.78±1.44	2.54±1.52	3.13±1.73	0.95	0.336	1.85±1.39	4.63±2.56	19.39	<0.001
P2(suspiciousness/persecutory ideas)	2.54±1.29	2.59±1.25	2.13±1.55	0.85	0.363	1.49±1.10	2.88±2.53	6.35	0.015
P3(grandiose ideas)	0.48±0.96	0.44±0.99	0.50±1.07	0.03	0.870	0.21±0.47	0.50±1.41	1.16	0.287
P4(perceptual abnormalities/hallucinations)	1.87±1.54	1.72±1.49	2.50±1.60	1.79	0.187	0.67±0.98	3.88±1.64	55.36	<0.001
P5(disorganized communication)	0.48±0.84	0.38±0.78	1.00±0.93	3.88	0.055	0.13±0.41	1.38±1.30	25.47	<0.001
N1(social anhedonia)	1.89±1.37	1.77±1.11	2.38±1.41	1.80	0.186	0.97±0.81	2.63±1.68	18.15	<0.001
N2(avolition)	1.17±1.07	1.23±1.11	1.50±0.76	0.43	0.518	0.51±0.79	2.00±1.31	18.48	<0.001
N3(expression of emotion)	1.03±1.16	0.82±1.07	2.00±1.19	7.73	0.008	0.41±0.55	2.25±1.91	27.37	<0.001
N4(experience of emotions and self)	0.81±1.04	0.54±0.82	1.75±1.03	13.21	0.001	0.31±0.52	2.13±1.64	33.81	<0.001
N5(ideational richness)	0.17±0.49	0.18±0.56	0.13±0.35	0.70	0.792	0.08±0.35	0.00±0.00	0.37	0.546
N6(occupational functioning)	1.44±1.77	1.49±1.92	1.50±1.77	0.00	0.986	0.79±1.22	2.50±1.77	11.08	0.002
D1(odd behavior of appearance)	0.57±1.04	0.44±0.79	1.25±1.49	5.06	0.029	0.21±0.66	2.37±1.92	33.30	<0.001
D2(bizarre thinking)	1.48±1.29	1.51±1.23	2.25±1.39	2.28	0.138	1.10±1.25	3.13±2.23	12.93	0.001
D3(trouble with focus and attention)	1.19±1.09	0.92±1.01	1.75±1.03	4.42	0.041	0.64±0.74	2.5±1.51	27.92	<0.001
D4(impairment in personal hygiene)	0.13±0.38	0.13±0.41	0.25±0.46	0.56	0.457	0.10±0.45	0.75±0.89	9.57	0.003
G1(sleep disturbance)	1.56±1.27	1.62±1.33	1.50±1.195	0.05	0.822	0.69±0.83	2.25±0.71	24.32	<0.001
G2(dysphoric mood)	2.43±1.17	2.41±1.14	3.13±1.13	2.62	0.113	1.21±0.95	3.50±1.19	35.46	<0.001
G3(motor disturbances)	0.27±0.57	0.21±0.57	0.50±0.53	1.81	0.185	0.05±0.22	0.50±0.53	15.43	<0.001
G4(impaired tolerance to normal stress)	1.37±1.34	1.56±1.46	1.50±1.41	0.01	0.910	0.85±1.20	2.50±1.60	11.18	0.002
GAF	65.37±12.22	65.05±13.84	65.63±9.80	0.01	0.912	73.31±15.31	57.63±11.27	7.49	0.009
MADRS	12.93±9.65	13.33±10.63	13.50±8.55	0.00	0.967	5.92±5.42	15.12±6.96	17.35	<0.001

*SOPS, Scale of Psychosis-Risk Symptoms; MADRS, Montgomery-Åsberg Depression Rating Scale; GAF, Global Assessment of Functioning; APS, Attenuated Psychosis Syndrome; APS-c, converters at 6 month follow up; APS-nc, non-converters at 6 month follow up*.

## Discussion

This is one of the few studies examining the conversion rate and prediction effect of APS individuals to full-blown psychosis in mainland China. The findings showed a 17% of conversion rate at a 6-month interval, which is quite consistent with the previous finding from the meta-analysis (Fusar-Poli et al., 2012a). Although the conversion rate of clinical high risk individuals is much higher than general population, most of them did not transfer to the psychosis. In the current study, 21 of APS individuals recovered at follow up and all the non-converters showed decreased scores on the most of the symptoms of the SOPS. A 10-year follow-up study of a group of individuals at ultra high risk for psychosis indicated that 65.1% of the individuals did not convert to frank psychosis, and the highest risk for transition was within the first 2 years (Nelson et al., 2013). Velthorst et al. (2011) found that clinical high risk individuals showed reduced scores on symptoms with the maximum in the first year and after 3 years, 75% of them recovered with no significant symptoms. We speculated that this phenomenon indicate that this is a state of the high risk and may fluctuate during the next few years dependent on individuals' resilience, social support as well as the brain development.

In individuals with APS, we examined the potential prediction effect of the symptoms and found that the negative symptom, esp. emotional experience could predict the transition at 6 month follow-up. Previous studies have shown that negative symptom could predict or increase the transition rate in the clinical high risk population (Demjaha et al., 2012; Piskulic et al., 2012; Nelson et al., 2013; Valmaggia et al., 2013), but the mechanism between the negative symptom and the outcome of high risk of psychosis is still not clear. Not only in the individuals with clinical high risk, have the abnormal emotional experiences also been found in the psychometrically defined schizotypal individuals (Shi et al., 2012; Wang et al., 2014). A recent meta-analysis demonstrated that individuals in the ultra high risk (UHR) of psychosis shown significant moderate deficits in affect recognition and affect discrimination in faces (van Donkersgoed et al., 2015). Another study shown that the profound deficits in emotion recognition at baseline combining with the suprathreshold of negative symptoms in individuals at clinical high risk for schizophrenia made a significant prediction for the conversion (Corcoran et al., 2015).

According to the staging model, during a prodromal period, the patient with APS may experience an increasingly oppressive internal emotional experience that might manifest as negative symptoms (Mishara, 2010). Therefore, emotional experience, expression and recognition might play a critical role in the development of APS. Taken together, the integrated consideration of both positive and negative symptoms, especially the emotional experience in SOPS may give a better solution on the prediction of the transition in a short time.

This study has several limitations. First, the sample size is small and since we recruited participants through a multicenter project, the difficulties of follow up made a high falling off rate. Second, the follow-up time was short and the effect of emotional expression on the long-time prognosis was not clear. Third, the measures in the current study focused on the clinical symptoms and did not measure the cognitive function or emotional processing, which may provide more information for better prediction model. Taken together, in the current study, we found a transition rate of 17% in Chinese APS individuals at 6 months interval and more important, emotional experiences and expression may play unique role in the prediction of the psychosis. It must be noted that, this was a preliminary finding that will have to be replicated in future studies.

## Author contributions

FC designed the study, administered the interview, analyzed the data and wrote up the first draft of the paper. XS, YY, NZ, HQ, LZ, ZJL, HL, ZL, and JL administered the interview and collected the data. XZ conceived the idea and supervised the study. YW and RC interpreted the findings and commented the first draft of the paper. All authors approved the final version of the paper.

### Conflict of interest statement

The authors declare that the research was conducted in the absence of any commercial or financial relationships that could be construed as a potential conflict of interest.
